# Acute Myeloid Leukemia with Basophilic Differentiation Transformed from Myelodysplastic Syndrome

**DOI:** 10.1155/2017/4695491

**Published:** 2017-03-27

**Authors:** Yasuhiro Tanaka, Atsushi Tanaka, Akiko Hashimoto, Kumiko Hayashi, Isaku Shinzato

**Affiliations:** ^1^Department of Hematology and Clinical Immunology, Nishi-Kobe Medical Center, Hyogo, Japan; ^2^Molecular Genetic Analysis Department, LSI Medience Corporation, Tokyo, Japan

## Abstract

Myelodysplastic syndrome (MDS) terminally transforms to acute myeloid leukemia (AML) or bone marrow failure syndrome, but acute myeloid leukemia with basophilic differentiation has been rarely reported. An 81-year-old man was referred to our department for further examination of intermittent fever and normocytic anemia during immunosuppressive treatment. Chromosomal analysis showed additional abnormalities involving chromosome 7. He was diagnosed as having MDS. At the time of diagnosis, basophils had not proliferated in the bone marrow. However, his anemia and thrombocytopenia rapidly worsened with the appearance of peripheral basophilia three months later. He was diagnosed as having AML with basophilic differentiation transformed from MDS. At that time, monosomy 7 was detected by chromosomal analysis. We found that basophils can be confirmed on the basis of the positivity for CD203c and CD294 by flow cytometric analysis. We also found by cytogenetic analysis that basophils were derived from myeloblasts. He refused any chemotherapy and became transfusion-dependent. He died nine months after the transformation. We should keep in mind that MDS could transform to AML with basophilic differentiation when peripheral basophilia in addition to myeloblasts develops in patients with MDS.

## 1. Introduction

Myelodysplastic syndrome (MDS) is a clonal disorder of hematopoietic stem cells and is characterized by bone marrow failure of normal hematopoietic cells and a dysplastic change of trilineage cells [1]. Some cases of MDS show basophilia or eosinophilia in the bone marrow, which indicates a poor prognosis [2, 3]. MDS often develops into acute leukemia, which is termed transformation. Many cases of MDS transform to acute myeloid leukemia (AML) [1], but AML with basophilic differentiation has been rarely reported. On the other hand, acute basophilic leukemia (ABL) was originally reported over one hundred years before and classified as a distinct entity by the World Health Organization (WHO) classification in 2008 [4].

We report a case of AML with basophilic differentiation transformed from MDS. To the best of our knowledge, this is the fifth case of ABL or AML with basophilic differentiation transformed from MDS. We also reviewed here the other four cases in the literature.

## 2. Case Presentation

An 81-year-old man was admitted to our hospital because of bilateral pitting edema of his legs for about one month in October 2015. He was operated on for prostate cancer twelve years ago and also for Vater papilla cancer five years ago. He did not receive any further treatment. Physical examinations revealed only bilateral pitting edema of his legs, and no abnormalities of his thorax and abdomen were found. He was diagnosed as having minimal change nephrotic syndrome (MCNS) on the basis of his renal biopsy results in November 2015. He started immunosuppressive treatment with cyclosporine (CsA) and prednisolone (PSL), and he achieved complete remission for MCNS about one month later. Thus, CsA was stopped and his PSL dose was gradually tapered. During immunosuppressive treatment, he occasionally had fever of more than 38°C and felt general malaise due to normocytic anemia, as shown by laboratory tests. Thus, he was referred to our department for further examinations. Laboratory examinations revealed the following: white blood cell (WBC) count, 4.2 × 10^9^/L with no abnormal cells; hemoglobin (Hb) level, 9.2 g/dL; platelet (Plt) count, 53.6 × 10^10^/L. A bone marrow smear showed 6.4% blasts and marked dysplasia of neutrophils and erythroid precursor cells. Flow cytometric analysis showed that the blasts were positive for CD13, CD33, CD34, CD56, and HLA-DR, which was consistent with myeloblasts. Chromosomal analysis by G banding showed additional add(7)(q22) in 2 out of 20 metaphase cells analyzed. These findings led to the diagnosis of myelodysplastic syndrome (MDS, refractory anemia with excess of blast-1) in accordance with the WHO 2008 classification [1] and MDS with multilineage dysplasia in accordance with the 2016 revision of WHO classification [6]. On the basis of revised international prognostic scoring system [7], he was classified as high risk. At that time, basophils were only 2.0% of all nucleated cells in the bone marrow. He received only one pack of red blood cell (RBC) transfusion as palliative therapy and felt better during oral PSL therapy.

**Figure 1:**
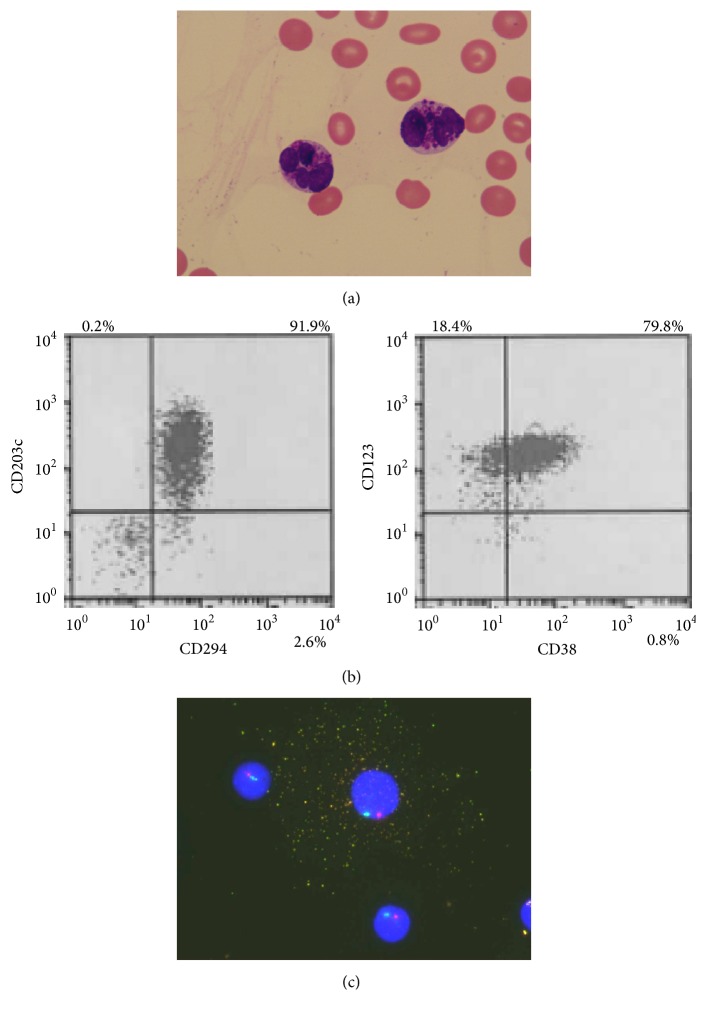
The characterization of basophils in peripheral blood. (a) Peripheral blood smear showed some medium- to large-sized abnormal cells with lobulated nucleus, and many basophilic granules were observed (May-Giemsa staining, original magnification, ×1000). (b) Flow cytometric analysis showed that abnormal cells were positive for CD38, CD123, CD203c, and CD294, which was consistent with basophils. (c) Dual-color FISH analysis using chromosome 7 probes showed that abnormal cell with many granules contained one red signal and one green signal, which indicated monosomy 7 (D7S486 probe, red signal; D7Z1 probe, green signal). Other cells without any granules also showed monosomy 7.

**Figure 2:**
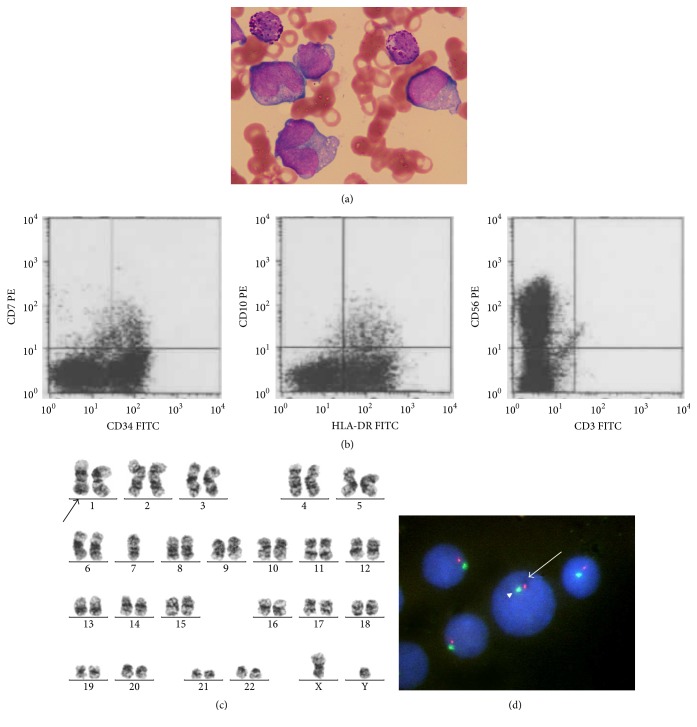
The characterization of myeloblasts in bone marrow. (a) Bone marrow smear showed some large-sized myeloblasts with clear nucleoli, basophilic cytoplasm, and some vacuoles. Some mature basophils were observed (May-Giemsa staining, original magnification, ×1000). (b) Flow cytometric analysis showed blasts positive for CD34, CD56, and HLA-DR. (c) Chromosomal analysis by G banding showed 45, XY, -7 [5]/45, idem, add(1)(q21) [4]/46, XY [2]. (d) Dual-color FISH analysis using chromosome 7 probes showed that myeloblasts contained one red signal and one green signal, which indicated monosomy 7 (D7S486 probe, red signal, arrow; D7Z1 probe, green signal, arrow head).

In February 2016, he complained again of general malaise. Some abnormal cells with many basophilic granules appeared in his peripheral blood and then gradually increased in number (Figure 1(a)). One month later, anemia and thrombocytopenia rapidly developed. Laboratory examinations revealed the following: WBC count, 2.5 × 10^9^/L with 2% myeloblasts and 47% abnormal cells; Hb level, 5.1 g/dL; Plt count, 4.8 × 10^10^/L. Flow cytometric analysis of abnormal cells showed that these cells were positive for CD11b, CD13, CD33, CD38, CD123, CD203c, and CD294 and negative for CD34 and HLA-DR, which was consistent with basophils (Figure 1(b)). Serum histamine (8.67 ng/ml; normal range, 0.15–1.23) and tryptase (6.6 *µ*g/L; normal range, 1.2–5.7) levels were slightly elevated. The bone marrow smear showed 30.4% myeloblasts and 16.8% mature basophils (Figure 2(a)). Flow cytometric analysis showed that the myeloblasts were positive for CD13, CD33, CD34, CD56, CD117, and HLA-DR (Figure 2(b)), which indicated the same phenotype as the blasts in the initial bone marrow examination. Fluorescence in situ hybridization (FISH) analysis showed neither fusion signals of the BCR and ABL genes nor those of the DEK and NUP214 genes. Chromosomal analysis by G banding showed monosomy 7 in 14 out of 20 metaphase cells and additional add(1)(q21) in 4 out of 20 metaphase cells analyzed (Figure 2(c)). Monosomy 7 was confirmed by FISH analysis (Figure 2(d)). The loss of chromosome 7 was found in myeloblasts and basophils of the bone marrow (Figure 1(c)), which showed that basophils were derived from myeloblasts. These findings indicated the diagnosis of AML with basophilic differentiation transformed from MDS. We did not check for basophilic granules by electron microscopy. He refused intensive chemotherapy and azacitidine treatment, and he was RBC-transfusion-dependent with 10 mg of oral PSL per day. In August 2016, he developed* Klebsiella pneumoniae* bacteremia complicated by solitary liver abscess. His bacteremia was treated with intravenous administration of tazobactam/piperacillin for six weeks. After his discharge, he had continued to receive regular RBC transfusion, but he suddenly died of bronchopneumonia at home in November 2016. An autopsy was not carried out. One week before his death, a laboratory examination showed the following: WBC count, 5.9 × 10^9^/L with 3% myeloblasts and 49% mature basophils; Hb level, 7.0 g/dL; Plt count, 4.0 × 10^10^/L. These findings did not change over nine months after the transformation.

## 3. Discussion

Here, we report the case of a patient with AML with basophilic differentiation transformed from MDS. We diagnosed him as having AML with basophilic differentiation on the basis of the results of flow cytometric analysis. More than 30% of blasts occupied the bone marrow and were positive for CD13, CD33, CD34, CD56, CD117, and HLA-DR, which was consistent with myeloblasts. Some mature basophils were found in the same bone marrow sample. On the other hand, in peripheral blood, many basophils were found. They were positive for CD11b, CD13, CD33, CD38, CD123, CD203c, and CD294 and negative for CD34 and HLA-DR. Both CD203c and CD294 are specific markers of basophils [8]. Thus, myeloblasts and basophils express different markers. We also showed by FISH analysis that basophils were derived from myeloblasts. FISH analysis showed the loss of chromosome 7 in myeloblasts and basophils in the bone marrow. This is the first case study by FISH analysis confirming that basophils were derived from myeloblasts. The origin of basophils in ABL has been shown by electron microscopy and chromosomal analysis by G banding thus far. These methods do not directly show the cell origin. Thus, FISH analysis was very useful for detecting the cell origin of basophils.

ABL is a very rare disease characterized by the proliferation of blasts containing basophilic granules in the cytoplasm. Although almost all cases of ABL were de novo [4], some cases of ABL transformed from myeloproliferative neoplasms (MPNs) have been reported thus far. The representative case of transformed ABL is the basophilic blast crisis of chronic myeloid leukemia (CML). In the case of the basophilic crisis of CML, although a very rare type of blast crisis, the basophils are derived from a CML clone, which is shown by chromosomal analysis by G banding [9]. Among MPNs except CML, only two cases of ABL transformed from MPNs were reported. One case was ABL transformed from essential thrombocytosis reported by Shah et al. [10], and the other was that transformed from primary myelofibrosis reported by Sugimoto et al. [11]. Thus, reports of cases of basophilic leukemia transformed from MPNs are relatively rare in the literature.

To the best of our knowledge, four other cases of ABL or AML with basophilic differentiation transformed from MDS have been reported (Table 1). Shirakawa et al. reported a case of a 52-year-old woman admitted for further examination of anemia [12]. She was diagnosed as having MDS (refractory anemia with excess of blast, RAEB). About two months later, her blood examinations showed increased severity of leukocytosis. Chromosomal analysis showed a complex karyotype including monosomy 7. She died of respiratory failure and bacteremia six months after the transformation. Yamagata et al. reported the case of a 52-year-old woman presenting with general malaise [13]. She was diagnosed as having MDS (refractory anemia, RA). Chromosomal analysis showed del(5)(q31q35). About two years later, bone marrow examination showed normocellularity with 42.8% blasts and 26.6% basophils. Chromosomal analysis revealed the same abnormalities as those in the initial diagnosis of MDS. She died of severe interstitial pneumonia two months after the transformation. Wells et al. reported the case of an 84-year-old woman presenting with general deterioration of her health [14]. She showed pancytopenia with peripheral blasts. Her neutrophils were almost dysplastic, and the blasts contained basophilic granules. She was diagnosed as having AML with myelodysplasia-related changes showing basophilic differentiation. Chromosomal analysis showed a complex karyotype including monosomy 7. This case was consistent with AML with myelodysplasia-related changes and basophilic differentiation transformed from MDS, which was not diagnosed previously. Bahmanyar and Chang reported the case of a 64-year-old man who presented with increased shortness of breath due to pancytopenia [5]. He was diagnosed as having MDS (refractory anemia with multilineage dysplasia, RCMD). Chromosomal analysis showed a complex karyotype including monosomy 7. Two months later, his MDS transformed to AML with myelodysplasia-related changes and prominent basophilic differentiation. Chromosomal analysis revealed the same abnormalities as those in the initial diagnosis of MDS. Clinical characteristics of ABL or AML with basophilic differentiation transformed from MDS were the very short interval (within six months) from the diagnosis of MDS to the transformation, monosomy 7 detected in 3 out of 5 cases by chromosomal analysis, and very poor outcome (i.e., death) in 3 out of 5 cases. These clinical features might be due to the monosomy 7 abnormality. Thus, we should keep in mind that MPNs could transform to ABL or AML with basophilic differentiation.

The normal range of basophils in the bone marrow was below 1% [15]. Our patient showed a slightly elevated basophil count in the bone marrow at the time of the diagnosis of MDS. Regarding the two cases in Japan previously reported [12, 13], we found that the percentage of basophils at the time of the diagnosis of MDS was slightly high in the bone marrow. We speculated that the increased percentage of basophils in the bone marrow at the time of the diagnosis of MDS would be a predictor of the transformation to ABL or AML with basophilic differentiation. The mechanism by which basophils differentiate from myeloblasts has not been clarified so far, and no mechanism has been proposed to the best of our knowledge. Collection of more cases of ABL or AML with basophilic differentiation transformed from MDS may clarify this mechanism.

In conclusion, we here reported the case of AML with basophilic differentiation transformed from MDS. The combination of flow cytometry and FISH analysis is useful easily for the diagnosis of AML with basophilic differentiation.

## Figures and Tables

**Table 1:** Previous reports of patients with acute basophilic leukemia or acute myeloid leukemia with basophilic differentiation transformed from myelodysplastic syndrome. M, male; F, female; RA, refractory anemia; RAEB, refractory anemia with excess blasts; RCMD, refractory cytopenia with multilineage dysplasia; ND, not described; IP, interstitial pneumonia.

Case	Age/sex	Diagnosis	Interval from diagnosis to transformation	Karyotype at transformation	Outcome	prognosis from transformation	Reference
1	52/F	RAEB	2 months	Complex karyotype including -7	Death of disease	6 months	Shirakawa et al., 1992
2	52/F	RA	2 years	del(5)(q31q35)	Death due to IP	2 months	Yamagata et al., 1995
3	84/F	ND	2 months	Complex karyotype including -7	ND	ND	Wells et al., 2014
4	64/M	RCMD	2 months	Complex karyotype including -7	ND	ND	Bahmanyar and Chang, 2016
5	81/M	RAEB-1	3 months	-7, add(1)(q21)	Death of bronchopneumonia	6 months	This case
